# Lessons learned from the development and implementation of a patient-reported outcome and experience measure (POEM) in an Australian glaucoma practice

**DOI:** 10.1186/s12886-019-1198-7

**Published:** 2019-08-22

**Authors:** Alison Fraenkel, Graham A. Lee, Stephen J Vincent, Roslyn A. Vincent, Rupert R. A. Bourne, Peter Shah

**Affiliations:** 1 City Eye Centre, 10/135 Wickham Terrace, Brisbane, Queensland 4000 Australia; 2 University of Queensland, Brisbane, Queensland Australia; 3 Queensland University of Technology, Brisbane, Australia; 4 Cambridge University Hospitals, Cambridge, UK; 5 University Hospitals Birmingham NHS Foundation Trust, Birmingham, UK; 6 Institute of Ophthalmology, University College London, London, UK; 7 Centre for Health & Social Care Improvement, University of Wolverhampton, Wolverhampton, UK

**Keywords:** Patient-reported outcome experience measure, Glaucoma, Patient engagement

## Abstract

**Background:**

A patient’s perception of how their glaucoma is managed will influence both adherence to their medication and outcome measures such as quality of life.

**Methods:**

Prospective consecutive study using a Glaucoma Patient-reported Outcome and Experience Measure (POEM) modified for an Australian ophthalmic private clinical practice setting. The Australian Glaucoma POEM consists of eight items related to the patient’s understanding of the diagnosis and management, acceptability of the treatment, whether they feel their glaucoma is getting worse, interfering with their daily life and concerns regarding loss of vision as well as addressing whether they feel safe under the care of their glaucoma team and how well their care is organised.

**Results:**

Two hundred and two patients (M:F 91:111) participated in the study. Mean ± standard deviation for subject age was 69 ± 13 years. Patient’s overall perception of their treatment and outcome was favourable. Younger patients felt their glaucoma interfered more with their daily lives and were more worried about losing vision from glaucoma. The greater the number of medications in use, the more they felt their glaucoma was getting worse and that glaucoma interfered with their daily lives. With all other variables accounted for by the multivariate linear model, female patients more strongly agreed that they understood their glaucoma diagnosis and glaucoma management. The patients with a severe visual defect in their worse eye, reported a greater perceived understanding of their glaucoma diagnosis and management and that they felt that glaucoma had a greater interference on their daily life. They were also more concerned about losing vision from glaucoma than their fellow glaucoma patients with less severe or no visual field deficit in the worse eye.

**Conclusions:**

The modified POEM demonstrates potential to capture the concerns of a practice’s glaucoma cohort with a view to enhancing the quality of glaucoma care delivered.

## Background

Glaucoma is a leading cause of visual impairment worldwide [1]. It has been dubbed “the silent thief of sight” because of its insidious onset and potential progression to complete blindness. Treatment needs to commence before patients notice any visual changes, which poses significant challenges to the patient in terms of adherence and tolerance [2–5]. The ocular medications required can be costly, irritate the ocular surface, have systemic side-effects and escalation to invasive surgical procedures may be necessary to prevent further progression of vision loss. This may all occur despite the patient still feeling asymptomatic [2–6]. Therefore a patient’s perception of how their disease is managed will influence both adherence and outcome measures such as quality of life [7]. Many measurement tools have been developed and validated for gauging patient reported outcomes and experiences for a wide scope of diseases [8–10]. For this study, the authors have adapted the Patient-reported Outcome and Experience Measure (POEM) that was developed by Somner et al. specifically for glaucoma, and applied it to a clinical practice setting [11].

**Fig. 1:**
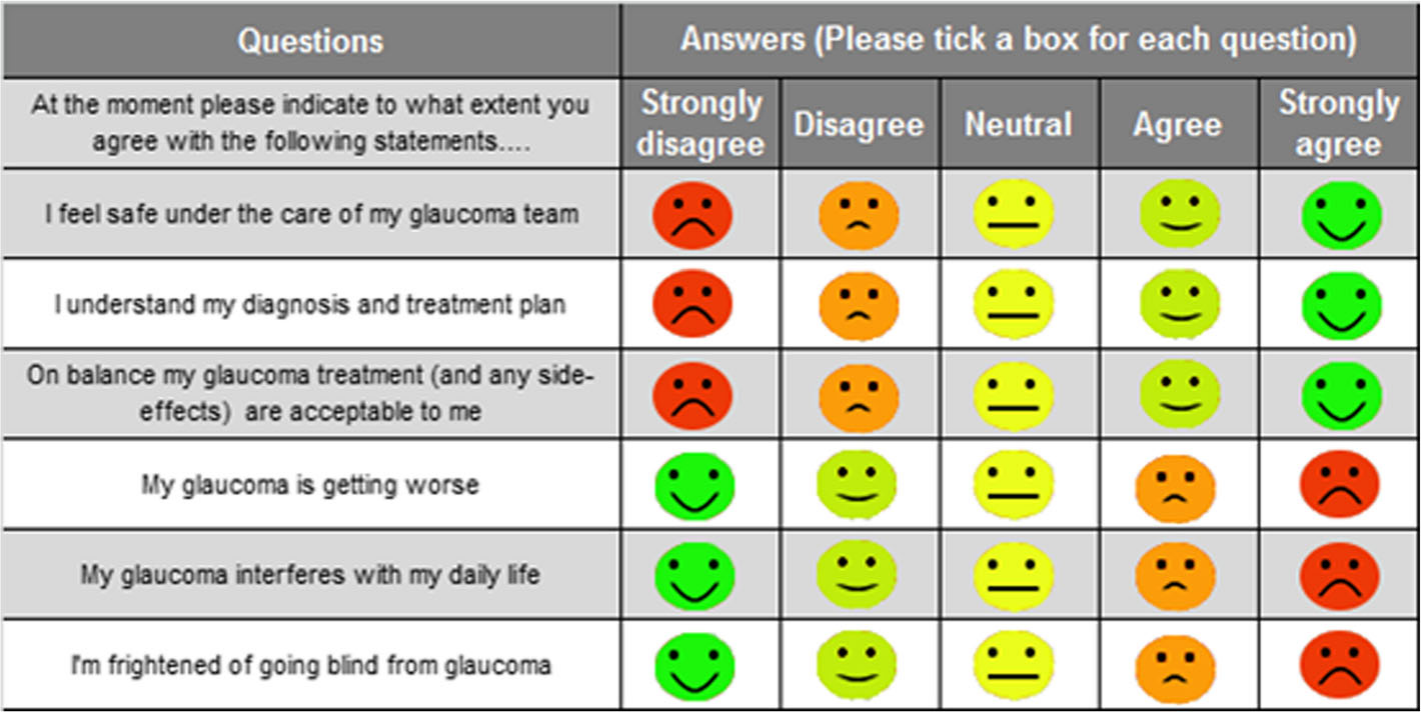
Glaucoma POEM developed in the UK by Somner et al [11]

The Glaucoma POEM is a novel six-item questionnaire that was developed from the “National Glaucoma Think Tank” event in the United Kingdom in 2012 (Fig. 1). This event was attended by 72 UK participants including 42 patients, 11 carers, 3 health workers and 15 ancillary staff. These participants were divided in to “focus groups” for discussion lead by 23 health care professionals including ophthalmologists. Discussion was based around what type of questions the participants would value in a glaucoma-specific Patient-reported Outcome Measure (PROM) or Patient-reported and Experience Measure (PREM). Narrative data from the discussions was subjected to thematic analysis with NVivo software, from which particular themes were extracted and sorted into PROM and PREM domains and subcategories. These were cross-referenced with existing generic and vision-specific PROMs and PREMs, of which none were found to sufficiently cover the domains and subcategories of importance. The six-question patient-generated POEM was derived from here, as a concise questionnaire that addressed the areas of glaucoma experience and outcome of greatest significance to the National Glaucoma Think Tank participants [11].

Prior to this study and to the authors knowledge, there has not been a published account of clinical implementation of the POEM. The POEM is designed to be a patient-generated implement and in the planning phases of application to a private ophthalmology setting in Australia, the POEM underwent minor modifications for enhanced situational and cultural appropriateness. The resulting survey comprised eight questions, the response for each to be marked on a Visual Analogue Scale (VAS), as opposed to six items each requiring ordinal categorical answer selection. We describe how implementation of the modified POEM is a useful tool in capturing patient perceived experiences and outcomes as a measure of quality in health care.

## Methods

**Fig. 2:**
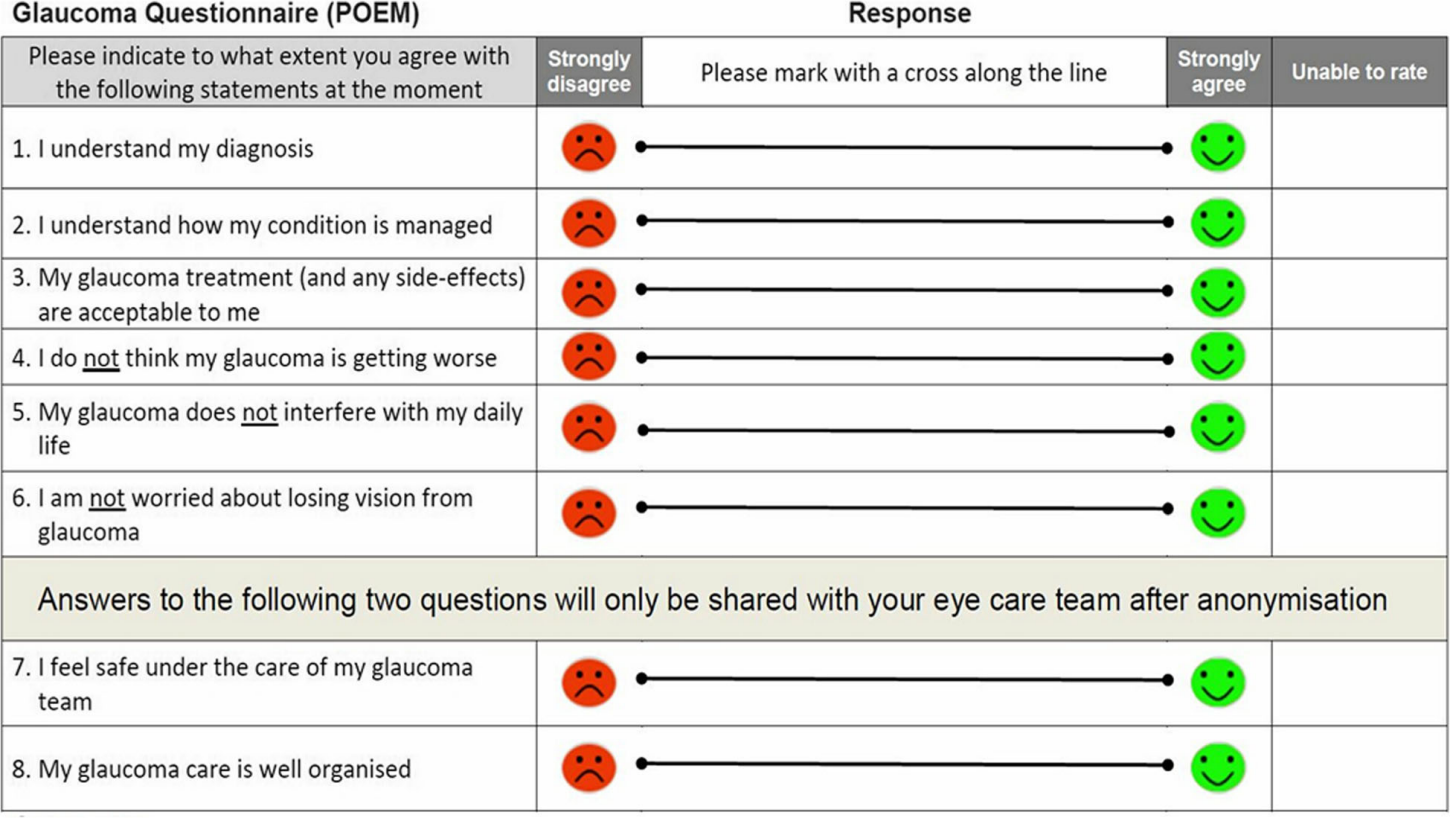
Australian Glaucoma POEM adapted from the UK POEM

In order to be implemented in an Australian private practice, the Glaucoma POEM underwent modification with input from ophthalmic consultants, an optometric statistician and an initial cohort of 25 patients. Patient input was voluntary and obtained during routine consultations. The Australian Glaucoma POEM consists of eight items related to the patient’s understanding of the diagnosis and management, acceptability of the treatment, whether they feel their glaucoma is getting worse, interfering with their daily life and concerns regarding loss of vision as well as addressing whether they feel safe under the care of their glaucoma team and how well their care is organised. Changes based on patient feedback included wording alteration, with substitution of the statement “I’m frightened of going blind from glaucoma” with “I am not worried about losing vision from glaucoma”, as the former was regarded by many of our cohort as “overly emotive”. In the UK Think Tank, fear of blindness was strongly expressed and the changes implemented in this Australian setting may reflect different disease complexity, models of care and case-mix of the populations studied. The Likert-type scale of the original Glaucoma POEM, shown in Fig. 1, was modified into a VAS in order to increase the quantitative analysis potential of the study. In an earlier modification of the POEM with the VAS, the orientation of the scale in the same direction to the original POEM, yielded a number of completion errors. It was decided that the scale should be “strongly disagree” to the left and “strongly agree” to the right, requiring the use of “not” within questions 4–6. Although potentially confusing, this change subsequently stopped the completion errors. The VAS is used by other patient-reported outcome surveys such as European-Quality of life 5-Dimensions score (EQ 5D) [12]. Figure 2 features the resulting modified POEM.

Two hundred and two consecutive glaucoma patients were recruited from three private practices affiliated with the City Eye Centre, Brisbane. Informed consent was obtained from all patients. This study followed the tenets of the Declaration of Helsinki and was approved by the University of Queensland Human Research Ethics Committee (approval number #2015000530). Patients were included if they had a known diagnosis of glaucoma, as confirmed by glaucoma subspecialist. They were excluded if they had other visually significant ocular comorbidities.

The patient surveys were cross-referenced with their files to collect data including patient age, gender, the number of glaucoma medications currently in use, number of prior glaucoma surgeries, best-corrected visual acuity (BCVA) in the worse eye on the day of survey completion and their current visual field results (Humphrey Field Analyser, Carl Zeiss AG, Oberkocken, Germany) (Table 1).

**Table 1:** Glaucoma cohort patient demographics and disease characteristics (*n* = 202)

Variable	
Age (years)	69 ± 13 (range 24–94)
Gender	Female 55%
	Male 45%
Laterality of disease	Unilateral 11%
	Bilateral 89%
Glaucoma type	POAG 91%
	Other 9%
Previous glaucoma surgery	None 58%
	One eye 22%
	Both eyes 20%
Other ocular surgery	None 38%
	Cataract surgery only 42%
	Anterior surgery ± cataract surgery 6%
	Vitreoretinal surgery ± cataract surgery 15%
Mean number of glaucoma medications	1.9 ± 1.1, range 0–4.5
Mean visual acuity (logMAR)	Worse eye: 0.5 ± 0.8, range − 0.2 to 3.5
	Better eye: 0.1 ± 0.3, range − 0.2 to 2.9
Visual field	Worse eye
	Normal 14%, Mild 26%, Moderate 23,37%
	Better eye
	Normal 29%, Mild 43%, Moderate 18%, Severe 10%

*POAG* Primary open angle glaucoma

The continuous patient variables for data analysis included patient age, BCVA (converted to LogMAR) in the worse eye and the number of glaucoma medications used in the worse eye. The categorical variables used for this study are listed below the continuous variables in Table 2. Previous glaucoma surgery included trabeculectomy, Molteno or Baerveldt tube insertion, selective laser trabeculoplasty, cyclodiode laser or laser peripheral iridotomy*.* Visual fields were categorically classified in accordance to the Hodapp-Anderson-Parrish grading scale where “mild” refers to a mean deviation (MD) of ≤ − 6 dB compared to an age matched population, “moderate” equals > − 6 dB but <− 12 dB, and severe is ≥ − 12 dB [13].

**Table 2:** Results for the linear multivariate model analysis of visual analogue scale outcomes with relation to patient demographic and disease variables

		B coefficient (95% CI lower limit, upper limit)							
Variable	Baseline category for categorical variables	Q1 I understand my diagnosis.	Q2 I understand how my condition is managed.	Q3 My glaucoma management (and any side effects) are acceptable to me.	Q4 I do not think my glaucoma is getting worse.	Q5 My glaucoma does not interfere with my daily life.	Q6 I am not worried about losing vision from glaucoma.	Q7 I feel safe under the care of my treating team.	Q8 My glaucoma care is well organised
Age		−0.2 (−0.4, −0.0)*	−0.2 (−0.4, 0.0)	0.1 (− 0.1, 0.4)	0.2 (− 0.2, 0.5)	0.4 (0.1, 0.8)#	0.5 (0.0, 0.9)*	0.1 (− 0.1, 0.2)	0.1 (− 0.1, 0.2)
BCVA worse eye (LogMAR)		−2 (− 5, 1)	−1 (− 4, 2)	− 1 (− 4, 3)	− 1 (− 6, 4)	−2 (− 7, 4)	− 1 (− 7, 6)	0 (− 2, 2)	0 (− 2, 1)
Number of glaucoma medications in worse eye		−1 (− 2, 1)	−1 (− 3, 1)	0 (− 2, 3)	−3 (− 12, 5)#	−6 (− 10, − 2)#	−4 (− 9, 0)	− 1 (− 2, 1)	−1 (− 2,1)
Gender	Male								
*Female*		5 (0, 10)*	6 (1, 10)#	1 (− 5, 7)	−1 (− 9, 7)	1 (− 7, 9)	2 (− 8, 12)	2 (− 1, 6)	1 (− 2, 4)
Glaucoma laterality	Unilateral								
*Bilateral*		−3 (− 12, 5)	−3 (− 11, 5)	4 (− 7, 14)	3 (− 12, 18)	9 (− 7, 25)	0.2 (− 19, 19)	−1 (− 7, 5)	−1 (− 7, 4)
Glaucoma type	POAG								
*Other glaucoma types*		− 1 (− 10, 8)	−3 (− 11, 5)	7 (− 4, 19)	8 (− 7, 23)	11 (− 5, 27)	0 (− 19, 20)	3 (− 4, 9)	2 (− 4, 7)
Previous glaucoma surgery	None								
*Unilateral*		−5 (− 12, 1)	−4 (− 11, 1)	− 2 (− 10, 6)	1 (− 10, 12)	−1 (− 13,11)	4 (− 10, 18)	0 (− 5, 5)	2 (− 2, 6)
*Bilateral*		−1 (− 8, 5)	− 7 (− 7, 5)	−8 (− 16, 0)*	−9 (− 20, 2)	−13 (− 25, − 2)*	−1 (− 15, 13)	1 (− 3, 6)	2 (− 2, 6)
Previous ocular surgery	None								
*Cataract*		0 (− 5, 6)	3 (− 2, 8)	0 (− 7, 7)	− 3 (− 12, 6)	−13 (− 23, − 3)#	5 (− 7, 16)	4 (− 2, 9)	0 (− 3, 4)
*Other ocular surgery*		6 (− 5,16)	7 (− 3, 17)	6 (− 7, 20)	1 (− 18, 19)	−17 (− 37, 2)	−21 (− 45, 2)	4 (− 1, 10)	2 (− 5, 8)
Visual field in worse eye	Normal								
*Mild*		1 (− 7, 8)	2 (− 5, 9)	1 (− 8, 11)	−4 (− 17, 9)	− 11 (− 24, 3)	−5 (− 22, 11)	0 (− 3, 4)	3 (− 2, 8)
*Moderate*		6 (− 2, 14)	7 (0, 13)	−1 (− 10, 9)	−8 (− 21, 6)	−10 (− 24, 4)	−11 (− 28, 6)	2 (− 6, 10)	3 (− 2, 8)
*Severe*		9 (2, 16)*	8 (1, 15)*	0 (− 10, 9)	− 19 (− 32, − 9)#	−18 (− 32, − 5)#	− 14 (− 31, 3)	−3 (− 8, 3)	5 (0, 10)

*- *p* < 0.05

# - *p* ≤ 0.01

*POAG* Primary open-angle glaucoma

The outcome data was the patients’ marked scores on the VAS, which was reported to whole numbers, with each VAS unit representing one mm on a 100 mm scale (i.e. a score between 0 and 100). The questions were designed so that the “strongly agree” end, hence VAS scores closer to 100, reflected more positively perceived experiences and outcomes. Statistical analysis was performed with IBM SPSS Statistics Software (Version 22; SPSS Inc., Chicago, IL, USA). Additionally, a linear multivariate model was created to predict the VAS outcome of each POEM question, with the patients’ demographic and disease characteristic variables as the predictors. The results of the multivariate linear analysis are reported in β coefficients and the corresponding 95% CI. For the continuous predictors including patient age, BCVA and number of glaucoma medications used, the β coefficient can be defined as the variance in VAS score, reported as a numerical increase or decrease in the VAS score, that can be accounted for by a one unit increase in the continuous variable. For the categorical predictors, the β coefficient represents the variance in VAS predicted when a subject belongs to the variable category, where the “baseline” category is considered to have no effect on the VAS score. The effect of a predictor, or variable, on the VAS score was considered to be statistically significant if its β coefficient had a *p*-value of <0.05.

## Results

Two hundred and two patients (M:F 91:111) participated in the study. Mean ± standard deviation for subject age was 69 ± 13 years. Severe visual field loss (MD ≥ -12 dB) was present in 75 (37%) (Table 1). The majority of the cohort (184 patients, 91%) had primary open angle glaucoma (POAG). Besides question 6 (“I don’t think my glaucoma is getting worse”), the questions yielded VAS with a strong right skew, indicating that patient’s overall perception of their treatment and outcome was favourable.

The results for the multivariate linear model are displayed in Table 2. For the association between patient age and response to question 1, regarding the perceived understanding of their glaucoma diagnosis, the multivariate linear modelling gave a statistically significant β coefficient of − 0.2 (95% CI − 0.4 to 0.0, *p* = 0.03). This means that with all other variables accounted for, an increase in age by one unit (1 year) explains a variance in − 0.2 on the VAS score, which would manifest as a mark 0.2 mm closer to the “strongly disagree” end of the VAS; that is the older the patient, the less their perceived understanding of their diagnosis. A similar association was evident for perceived understanding of glaucoma management, but this did not reach statistical significance (*p* = 0.08).

### Age

There was a statistically significant association between patient age and perceived disease outcome as per questions 5 and 6. Younger patients felt their glaucoma interfered more with their daily lives (β coefficient = 0.4, 95% CI - 0.1 to 0.8, *p* = 0.01) and were more worried about losing vision from glaucoma (β coefficient = 0.5, 95% CI -0.0 to 0.9, *p* = 0.04). No other significant associations were apparent between age and the POEM responses.

### BCVA in the worse eye

Although not reaching statistical significance, the more severe loss of BCVA in the worse eye was consistently associated with less favourable perceptions, for questions 1 to 6. This variable however, did not adversely affect the patient experience of the treating team, in terms of feeling safe under the team’s care or in the organisation of care (β coefficient = 0, 95% CI − 2 to 2, *p* > 0.05 and β coefficient 0, 95% CI − 2 to 1, *p* > 0.05 respectively).

### Number of medications in the worse eye

A greater number of medications used in the worse eye was associated with less favourable responses for most of the POEM questions, but only significantly for questions 4 and 5. The greater the number of medications in use, the more they felt their glaucoma was getting worse (β coefficient = 3, 95% CI − 12 to 5, *p* = 0.005) and that glaucoma interfered with their daily lives (β coefficient = 6, 95% CI − 10 to − 2, *p* = 0.002).

### Gender

With all other variables accounted for by the multivariate linear model, female patients more strongly agreed that they understood their glaucoma diagnosis (β coefficient = 5, 95% CI - 0 to 10, *p* = 0.05) and glaucoma management (β coefficient = 6, 95% CI - 1 to 10, *p* = 0.01).

### Previous glaucoma surgery

Compared to those patients with no history of glaucoma surgery, those who had had bilateral glaucoma surgery indicated that their glaucoma management was less acceptable to them (β coefficient = 8, CI − 16 to 0, *p* = 0.04) and that glaucoma interfered more with their daily lives (β coefficient = 13, 95% CI − 25 to − 2, *p* = 0.03).

### Other ocular surgery

Patients who had any other type of ocular surgery felt glaucoma interfered with their daily lives as indicated by the negative coefficients for all sub-categories. Only the β coefficient for previous cataract surgery was statistically significant at − 13 (95% CI − 23 to − 3, *p* = 0.01).

### Visual field in the worse eye

The patients with a severe visual defect in their worse eye, reported a greater perceived understanding of their glaucoma diagnosis and management (β coefficient = 9, 95% CI - 2 to 16, *p* = 0.02 and β coefficient = 8. 95% CI - 1 to 15, p = 0.03 respectively). and that they felt that glaucoma had a greater interference on their daily life (β coefficient = − 19, − 32 to − 9, p = 0.01). They were also more concerned about losing vision from glaucoma (β coefficient − 18, 95% CI − 32 to − 5) than their fellow glaucoma patients with less severe or no visual field deficit in the worse eye.

## Discussion

There are a number of patient-reported outcome and experience measures that have been applied to glaucoma [11, 14–16]. The advantage of many of these pre-existing tools is that they have been externally validated for reliability and consistency. However, there are multiple factors that make the available tools suboptimal for routine clinical implementation in glaucoma [11]. A number have been designed for research purposes, with lengthy questionnaires, requiring administration by trained personnel [11, 15, 17]. Other tools are shorter, comprehensive and validated for a variety of different medical conditions, such as the howRUTM and the EQ 5DR, however these have limited clinician accessibility due to patents and cost [11, 18]. Overall, there is not an established tool that has been optimised for use in routine glaucoma clinical practice [11]. We propose the modified POEM as a novel measure for gauging glaucoma patients’ perceived outcomes and experiences in one comprehensive and clinically applicable tool.

The modified POEM has potential to be useful for glaucoma patient feedback in clinical practice because it is designed specifically for this purpose, and has been developed with a “patient-generated” approach. Construction of the original POEM from focus groups means that the survey addresses topics that are most pertinent to glaucoma patients, and it can be customised to be more culturally appropriate as we have done for this study [11]. Like those involved in the original POEM conception, the patients involved in this POEM modification and implementation demonstrated willingness to participate and perceived it as a positive exercise in patient involvement. From a clinician’s perspective, the modified POEM is practical for daily clinical use because it is simple, short and provides quantitative data for comprehensive analysis. This study demonstrates the detailed insight the POEM can deliver on a patient cohort’s perception of their glaucoma management and outcomes.

The right skew of almost all VAS scores with medians above 85 reflects an overall positive response to perceived glaucoma experience and outcomes. Question 6 was the only item with a median VAS score of below 50 and had the widest interquartile range, reflecting a wide spread of concern about losing vision from glaucoma. Questions 4 and 5 demonstrate the next largest quartile ranges. It can be noted that Questions 4–6 are related to glaucoma outcome. These results are reflective of the fact that the severity of glaucoma can vary greatly between patients and at different points of the disease process, ranging from imperceptible vision loss to blindness. Questions 7 and 8 relate to patients’ perception of their glaucoma experience specific to the treating team, with the results suggesting that patients in this cohort feel secure regardless of demographic or disease variables. The finding that previous cataract surgery is associated with the perception that glaucoma affects their daily lives more seems counterintuitive. One possibility is that cataract surgery is often associated with improved vision, but if there is significant glaucoma nerve damage, the vision may not be significantly improved, with resulting disappointment regarding the visual outcome. The non-linear relationship between decline in vision-related quality of life (VRQL) and worsening of the visual field is well documented. In earlier stages of vision loss, there is slow decline in the VRQL, however more rapid phase of change once the mean deviation in the better eye worsens beyond -15 dB [3].

The responses to questions 1 and 2 demonstrate that overall, this patient cohort strongly agreed with the statement that they understand their glaucoma diagnosis and management. Greater patient understanding of a medical condition is linked with greater treatment adherence, increased patient satisfaction and reduced anxiety [8, 9, 17]. The latter was shown in a recent Australian randomized trial conducted by Skalicky et al. involving 101 newly-diagnosed glaucoma patients [19]. Half the patients received standard care and counselling by their ophthalmologists whilst the other half received an additional telephone counselling session and glaucoma information mail out. The second group was found to have a statistically significant lower level of anxiety (− 0.60 logits, *p* = 0.02). They also demonstrated an increase in knowledge to the control group however this was not statistically significant (0.45 logits, *p* = 0.07) [19]. The approach for this study, was to assess the patients’ perceived level of knowledge, as opposed to their actual level of knowledge.

This study suggested that the older the patient, the less they understood their glaucoma diagnosis and how it was managed (*p* = 0.03 and 0.08 respectively). However, they did feel that the condition had less impact on their daily lives and that they were less worried about losing vision from glaucoma (*p* = 0.01 and *p* = 0.04). Younger patients are more likely to be concerned about the potential for loss of income-earning capacity and threat to their quality of life over many years. They may subsequently be more motivated to ask questions regarding prognosis and seek resources about glaucoma. These findings are consistent with those from Odberg et al’s study involving 589 glaucoma subjects in Norway who completed an in-depth survey investigating the impact of glaucoma on quality of life [17]. Patients younger than the age of 60 expressed a greater demand for information and greater anxiety about their prognosis. This suggests that younger patients may require more consultation time and education materials. Newly diagnosed POAG patients have been reported to understand their condition better than those diagnosed more than 2 years, however longer diagnosed patients understood better their condition to be long term [20].

The findings of this study are potentially limited to this select private practice population of Caucasian, well-educated and higher socio-economic patients. These patients were appreciative for the opportunity to complete the study as they understood the importance of feedback, that would ultimately improve their own care. Further studies using the POEM, would need to be adapted accordingly to other population groups, with the comparative results of great interest, for example in a public hospital outpatient setting. Other limitations of the modified POEM for clinical implementation include the resources required in the administration of a paper survey and recording the results. As noted by Øvretveit et al., a successful patient perception measure should be simple to integrate in to clinical workflow [9]. Some of these limitations could be overcome by adaptation of the paper survey in to an electronic format, potentially accessible via a smartphone device. This would facilitate automatic data collection and streamline data analysis. The duration of the patient’s glaucoma may also have had an influence on the POEM responses, however was not addressed in this study. With further use of the POEM on a larger glaucoma population, the validity, consistency and reliability of the tool could be better evaluated. Follow up administration of the POEM to the same population over time also could detect whether changes to the system have been effective in improving the quality of care [21].

## Conclusion

The modified glaucoma POEM can provide detailed information about a glaucoma cohort’s perception towards their disease management experience and outcomes and demonstrates advantages in implementation compared to existing tools. It has potential to be useful for a means of patient engagement and improvement in the delivery of quality health care.

## Data Availability

The datasets used and/or analysed during the current study are available from the corresponding author on reasonable request.

## Abbreviations

BCVA: Best-corrected visual acuity
dB: Decibel
MD: Mean deviation
POEM: Patient-reported Outcome and Experience Measure
PREM: Patient-reported and Experience Measure
PROM: Patient-reported Outcome Measure
VAS: Visual Analogue Scale
